# Adsorption of Biomineralization Protein Mms6 on Magnetite (Fe_3_O_4_) Nanoparticles

**DOI:** 10.3390/ijms23105554

**Published:** 2022-05-16

**Authors:** Kosuke Arai, Satoshi Murata, Taifeng Wang, Wataru Yoshimura, Mayumi Oda-Tokuhisa, Tadashi Matsunaga, David Kisailus, Atsushi Arakaki

**Affiliations:** 1 Division of Biotechnology and Life Science, Institute of Engineering, Tokyo University of Agriculture and Technology, 2-24-16 Naka-cho, Tokyo 184-8588, Japan; kouok-1030.kej-332@ezweb.ne.jp (K.A.); s-murata@st.go.tuat.ac.jp (S.M.); yosh.wasabi@gmail.com (W.Y.); mayumi.tokuhisa@gmail.com (M.O.-T.); tmatsuna@cc.tuat.ac.jp (T.M.); 2 Department of Materials Science and Engineering, University of California at Irvine, Irvine, CA 92697, USA; taifengw@uci.edu (T.W.); dkisailu@uci.edu (D.K.); 3 Japan Agency for Marine-Earth Science and Technology (JAMSTEC), 2-15 Natsushima-cho, Yokosuka 237-0061, Japan

**Keywords:** protein adsorption, metal oxide, nanoparticle, biomineralization, magnetotactic bacteria

## Abstract

Biomineralization is an elaborate process that controls the deposition of inorganic materials in living organisms with the aid of associated proteins. Magnetotactic bacteria mineralize magnetite (Fe_3_O_4_) nanoparticles with finely tuned morphologies in their cells. Mms6, a magnetosome membrane specific (Mms) protein isolated from the surfaces of bacterial magnetite nanoparticles, plays an important role in regulating the magnetite crystal morphology. Although the binding ability of Mms6 to magnetite nanoparticles has been speculated, the interactions between Mms6 and magnetite crystals have not been elucidated thus far. Here, we show a direct adsorption ability of Mms6 on magnetite nanoparticles in vitro. An adsorption isotherm indicates that Mms6 has a high adsorption affinity (Kd = 9.52 µM) to magnetite nanoparticles. In addition, Mms6 also demonstrated adsorption on other inorganic nanoparticles such as titanium oxide, zinc oxide, and hydroxyapatite. Therefore, Mms6 can potentially be utilized for the bioconjugation of functional proteins to inorganic material surfaces to modulate inorganic nanoparticles for biomedical and medicinal applications.

## 1. Introduction

Nature has evolved sophisticated strategies via biomineralization processes to synthesize functional inorganic materials [1,2,3,4,5,6,7,8,9,10]. Most biomineralization processes occur under ambient physiological conditions, often templated or guided by proteins. Specific proteins involved in biomineralization systems such as bone [11], teeth [12], and eggshells [13] have been identified from the surfaces of biominerals. The proteins promote or inhibit nucleation and crystal growth by interacting with ions, clusters, crystals, or the intermediates of inorganic phases, and have also been used to synthesize non-biogenic inorganic materials [14,15,16,17]. Although the mechanisms of the interactions of some proteins and their functions in biomineralization have been elucidated using in vitro mineral binding analyses [12,13,18], the molecular basis of biomineralization has largely been unexplored.

Magnetotactic bacteria synthesize intracellular magnetic nanoparticles consisting of magnetite (Fe_3_O_4_) [19]. In general, the synthesis of magnetite nanoparticles in magnetotactic bacteria occurs within internal lipid vesicles. The particle size and shape formed within the vesicle are specific to the bacterial cell types or species. Some cells produce uncommon crystal shapes such as elongated-octahedra and bullet shapes [20,21], which cannot be synthesized using a synthetic chemical approach. Therefore, the shape control mechanism has attracted much attention in the field of materials development and magnetic recording industries [22,23]. *Magnetospirillum magneticum* AMB-1 synthesizes magnetite nanoparticles of approximately 40 nm in diameter with a cubo-octahedral shape [24]. In fact, Mms5, Mms6, Mms7, and Mms13 belong to a group of biomineralization proteins specific to magnetotactic bacteria, which play a major role in the morphological control of both octahedral and cubo-octahedral magnetite nanoparticles [25]. In addition, these proteins show common features in their amino acid sequences such as the N-terminal regions containing glycine- and leucine-repetitive sequences, which are most likely transmembrane domains; the C-terminal region is rich in acidic amino acids (i.e., aspartic and glutamic acid) [26]. A comparative functional analysis of these four proteins in living bacterial cells conducted by establishing each gene deletion mutant revealed that the four genes are involved in magnetite crystal growth and control the geometries of the crystal surface structures [25,27].

Mms6 is the most studied protein in magnetite biomineralization systems and the iron-binding ability in its C-terminal region is reported for both ferrous (Fe^2+^) and ferric ions (Fe^3+^) [26,28,29,30,31]. The protein changes its conformation upon iron binding [29]. In in vitro chemical syntheses of magnetite nanoparticles, cubo-octahedral shaped crystals consisting of (100) and (111) facets were formed in the presence of Mms6, while octahedral crystals consisting of (111) facets were formed in its absence [32]. The C-terminal region alone can contribute to control the crystal shape of magnetite [33,34]. These observations suggest that Mms6 directly binds to magnetite crystal surfaces; most probably, the acidic peptide domain interacts with the (100) facets and inhibits crystal growth. However, the binding ability of Mms6 to magnetite crystals has not been elucidated yet.

In this study, we investigated the binding properties of Mms6 on magnetite nanoparticles to understand the potential adsorption mechanisms and function of Mms6 with respect to controlling the magnetite crystal shapes. Moreover, adsorption studies have also been conducted using inorganic oxide materials, which can be widely used in the biomedical and biotechnology fields. The findings of this study contribute to a fundamental understanding of the magnetite biomineralization mechanisms and protein recognition of inorganic surfaces. Moreover, the binding ability can be used to modify magnetite surfaces with functional molecules using Mms6 as an anchor. The prepared magnetic materials may be useful in separation technologies, sensors, and delivery applications in biotechnology.

## 2. Results and Discussion

**Figure 1:**
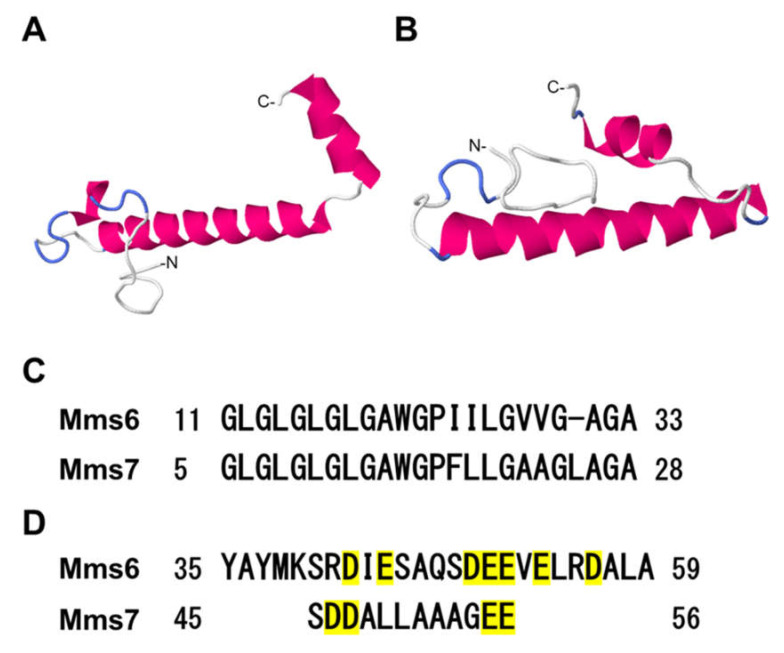
Characteristic amino acid sequences in the Mms proteins. The structure prediction of Mms6 (**A**) and Mms7 **(B**) by RaptorX software. (**C**) The sequence alignment of the N-terminal hydrophobic region. (**D**) The sequence alignment of the C-terminal hydrophilic region; acidic amino acids are highlighted in yellow.

Mms6 and Mms7 conserve a common secondary structure consisting of a hydrophobic N-terminal and hydrophilic C-terminal side. A tertiary structure prediction using the RaptorX web server [35] showed that both proteins had either long or short α-helix segments at the N-terminus and the C-terminus, respectively (Figure 1A,B). The N-terminal hydrophobic domain was predicted to be part of a transmembrane region (Figure 1C). In the hydrophilic domain, there were seven and four acidic amino acids in Mms6 and Mms7, respectively (Figure 1D). The adsorption abilities of these two proteins were examined to understand its mineral binding mechanism. In order to help elucidate this mechanism, α-synuclein and bovine serum albumin (BSA), with different isoelectric points but secondary structures and molecular weights (MW) similar to Mms6 and Mms7, were investigated (Table 1). α-Synuclein is a protein found in the neurons of the human brain and has a series of hydrophilic amino acid residues at the C-terminus, similar to Mms6 and Mms7 (Table S1). Unlike Mms6 and Mms7, α-synuclein has two α-helices at the N-terminus but no secondary structures at the C-terminus, and was thus used to examine the role of the C-terminal hydrophilic and α-helix regions in the adsorption of magnetite nanoparticles. In addition, previous studies with BSA indicated the role of electrostatic interactions in the adsorption mechanism [36] as it has multiple acidic amino acid residues, comprising 18.7% of its amino acid sequence. The acidic amino acid content of BSA is similar to that of Mms6 and Mms7 (11.9% and 9.6%, respectively); however, BSA does not have a hydrophilic region with acidic amino acid residues at its C-terminus. Therefore, BSA was also used to assess the adsorption ability of the magnetite biomineralization proteins (Mms6 and Mms7).

### 2.1. Adsorption of Proteins on Magnetite Nanoparticles

**Figure 2:**
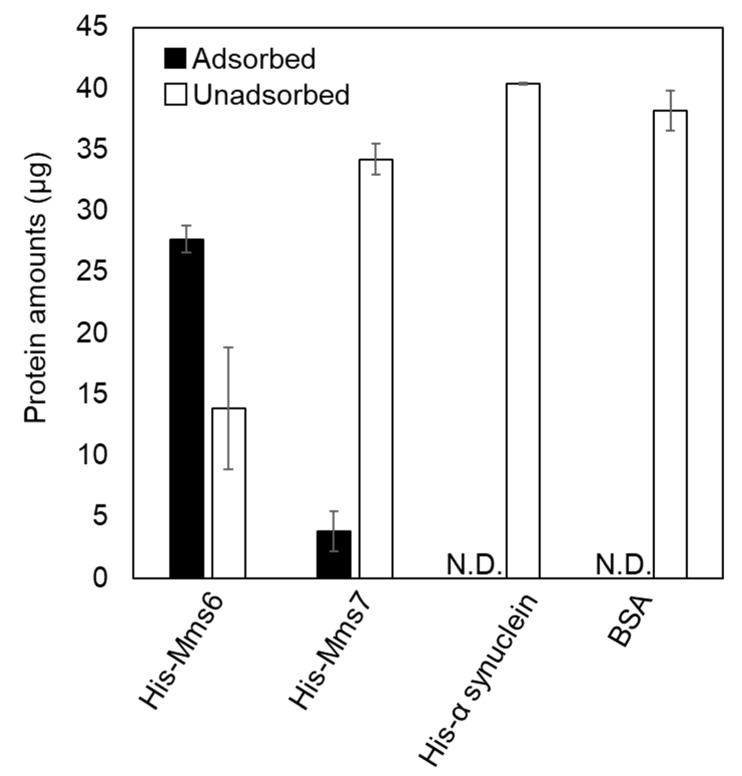
The adsorption assay of different proteins on magnetite nanoparticles. Adsorbed (black bar) or unadsorbed (white bar) protein amounts of His-Mms6, His-Mms7, His-α synuclein, BSA, cytochrome *c*, and lysozyme. A solution containing 40 µg of protein was added to 2.5 mg of the particles. N.D.: not detected.

To investigate the adsorption properties of the magnetite biomineralization proteins on magnetite crystals, an adsorption assay using 40.0 µg of proteins and 2.5 mg of spherical magnetite nanoparticles with a mean diameter of 35.1 ± 12 nm (SP35) was performed. Recombinant proteins eluted from Ni-NTA agarose resin by phosphate buffer (pH 8.0) were utilized for the adsorption assays. The pH selected was close to the estimated value of the magnetite formation condition within the internal vesicle of *M. magneticum* AMB-1 [37]. To determine the adsorbed protein amount, proteins were desorbed by boiling in 1% SDS solution. Then, the protein concentration was determined by the BCA assay, and was defined as the adsorbed amount. As a 1% SDS treatment with boiling is considered to desorb proteins completely from nanoparticles, the total amount of protein in the adsorbed and unadsorbed fractions was close to 40 µg. Among the proteins investigated in the present study, histidine tagged (His-) Mms6 showed the highest adsorption ability (ca. 27.7 µg, Figure 2) while only 3.8 µg of His-Mms7 was adsorbed onto the magnetite nanoparticles. Since the amount of protein detected in the adsorbed fraction was less than 0 µg (not detected by BCA assay), His-α-synuclein and BSA were considered to have no adsorption ability on the magnetite nanoparticles.

The electrostatic interactions between the carboxylic groups of acidic amino acid residues are considered to be responsible for the adsorption of proteins on inorganic materials [36,38,39]. In this study, both Mms6 and Mms7 proteins contained acidic amino acids and showed an adsorption ability to magnetite nanoparticles, consistent with previous findings. However, although BSA had a high acidic amino acid content (18.7%), it showed no significant adsorption on the magnetite nanoparticles. This is likely because the acidic amino acid residues in BSA were distributed throughout the entire sequence (i.e., delocalized), whereas those in the magnetite biomineralization proteins were localized at the hydrophilic C-terminus, providing a highly concentrated negative charge (Figure 1D). Furthermore, magnetite biomineralization proteins had both long and short α-helix secondary structures at the N-terminus and the C-terminus, respectively. Adsorption studies on statherin, an enamel pellicle protein that inhibits hydroxyapatite nucleation and growth [40], also indicated the importance of α-helices in protein-inorganic adsorption. Therefore, Mms6 and statherin have similar sequential and structural features. In this study, recombinant proteins were conjugated with a histidine tag for purification from the bacterial cell lysates. Because His-tag has a high binding affinity to metal ions such as Ni^2+^ and Co^2+^, it may contribute to the adsorption of magnetite nanoparticles. However, His-α-synuclein did not show any adsorption, suggesting that the adsorption of His-Mms6 and His-Mms7 was mediated by their conserved amino acid sequences, and not the histidine tag.

It is worthy to note that the theoretical pI of His-Mms6, estimated from its amino acid sequence (Table 1) is 6.0, while the pI of magnetite is between 6.5 and 7.3, depending on the synthesis conditions and ionic strength [41]. Under the pH conditions (i.e., pH 8.0) investigated in this study, both the His-Mms6 and magnetite nanoparticles were negatively charged, generating an electrostatic repulsion between the proteins and particles. However, our data clearly showed the binding of His-Mms6 to the magnetite nanoparticles. A similar protein adsorption to the magnetite nanoparticles under alkaline conditions (pH 8.1) was observed in BSA (pI 4.9) with carbodiimide [42]. Although electrostatic interaction is one of the driving forces for the adsorption, the binding observed in this study should involve other mechanisms. At pH ~8, the magnitude of the charge on the magnetite particle surface should actually not be very large. As such, electrostatic repulsive forces may not be sufficient to provide a barrier to adsorption [43]. In colloidal systems, significant charges (>40 mV) as measured by the zeta potential will be required to maintain the electrostatic dispersion [44,45]. At the pH value used in this study, it is unlikely that the charge on the magnetite will be sufficient to prevent the localized aspartic acid groups (which should be highly negatively charged due to the pI of ~3) from binding to the magnetite surface. The ligand substitution reaction between the carboxylic groups and aquo/hydroxyl ions is known as the principal driving force for the adsorption of organic acids on iron oxides in soil [46]. This reaction through the carboxylic groups of His-Mms6 can also be involved in the protein adsorption to magnetite nanoparticles. The hydrophobic region of His-Mms6 may also partially contribute to the adsorption. An adsorption study of peptides on silica nanoparticles showed the involvement of hydrophobic interactions in an organic–inorganic adsorption mechanism [47].

### 2.2. Adsorption Affinity of Mms6 to Magnetite Nanoparticle

**Figure 3:**
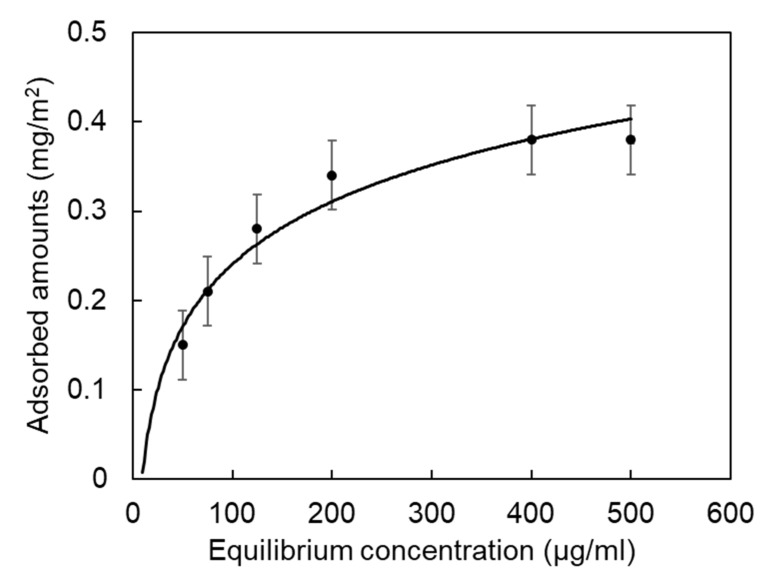
Adsorption isotherm for the His-Mms6 protein interacting with the magnetite nanoparticles. The dissociation constant obtained from the curve fitting was Kd = 9.52 µM.

To understand the adsorption affinity of Mms6 to magnetite nanoparticles, His-Mms6 (10.0−100.0 µg) was added to 2.5 mg of SP35 and the amount of adsorbed protein was measured. The results indicated that the adsorption of His-Mms6 on SP35 was saturated at approximately 30 µg of protein. Based on this result, an adsorption isotherm was constructed (Figure 3) and the dissociation constant (Kd) for the adsorption of His-Mms6 on SP35 was calculated to be 9.52 µM. The Kd values for the binding of Mms6 to ferric and ferrous ions were found to be between 0.43 to 6.0 µM, respectively [30,48]. The adsorption affinity of His-Mms6 to the magnetite nanoparticles was comparable to that of Mms6 to the iron ions. In addition, the adsorption affinity of Mms6 was compared to that of other biomineralization proteins such as amelogenin, which plays a role in the biomineralization of teeth [49,50]. The Kd values of statherin and amelogenin on hydroxyapatite were 111 µM [51] and 507 nM [52], respectively. Therefore, the adsorption affinity of His-Mms6 to the magnetite nanoparticles was comparable to, or higher than, that of the other biomineralization proteins, suggesting that His-Mms6 has a significant affinity to magnetite nanoparticles.

As described earlier, a hydrophilic region with a high concentration of acidic amino acids contributes to the high adsorption affinity of Mms6 to magnetite nanoparticles. Magnetite biomineralization proteins such as Mms6 are amphiphilic molecules consisting of hydrophobic and hydrophilic regions, and can self-assemble into micelle-like structures of approximately 20 molecules in solution [30]. Micelle formation results in the outward orientation of the hydrophilic region, resulting in a high density of acidic amino acid residues on the micelle surface. Therefore, the surface of the magnetite biomineralization proteins is strongly charged compared to that of other soluble proteins, which favors strong electrostatic interactions with the magnetite nanoparticles. Consistently, His-Mms6 showed a high adsorption affinity on the magnetite nanoparticles while that of His-Mms7 was lower. In the hydrophilic C-terminus of His-Mms6, there was a high density (ca. 7) of acidic amino acids, whereas a similar region in His-Mms7 only contained four acidic amino acid residues. Therefore, the surface charge density on Mms7 was lower than that on Mms6, which may account for its lower adsorption ability. Another difference between Mms6 and Mms7 was the presence of acidic amino acids in a short α-helix domain of the C-terminal region (Figure 1A,B). In the presence of ferrous ions, the conversion of an α-helix to a β-sheet secondary structure (Figure S1) supports the role of having an α-helix region within His-Mms6 to promote the adsorption on magnetite nanoparticles. In His-Mms6, the acidic amino acids at the C-terminus were predicted to form an α-helix and in the presence of ferrous ions, a conversion to a β-sheet secondary structure (Figure S1) was predicted, suggesting that this region is converted to a β-sheet structure, enabling the effective adsorption to magnetite. Conversely, the α-helix C-terminus region of His-Mms7 did not contain any acidic amino acid residues, thus did not show as strong an adsorption.

### 2.3. Electron Microscopic Observation of Protein-Nanoparticle Conjugates

**Figure 4:**
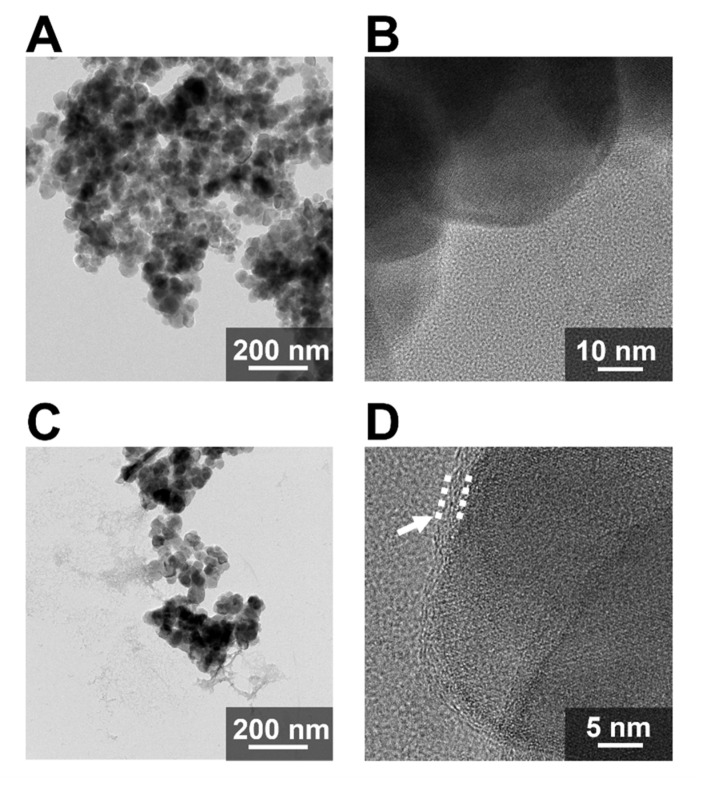
The TEM image of the magnetite nanoparticles in the absence (**A**) or presence (**C**) of the His-Mms6 protein. A single magnetite nanoparticle observed using HR-TEM in the absence (**B**) or presence (**D**) of the His-Mms6 protein. The white arrow indicates the protein corona.

To demonstrate the presence of His-Mms6 on the surfaces of the magnetite nanoparticles, transmission electron microscope (TEM) observations of the magnetite nanoparticles (SP35) in the presence of His-Mms6 were conducted. The TEM images showed the presence of disordered, low-contrast materials surrounding aggregates of nanoparticles (Figure 4A,C). Previous studies regarding the adsorption of proteins on inorganic nanoparticles indicated the formation of a protein adsorption layer (i.e., protein corona) [39,53]. High-resolution transmission electron microscopy (HR-TEM) of the stained samples showed a similar structure with a thickness of 2.4 ± 0.7 nm, suggesting the presence of His-Mms6 on the nanoparticle surface (Figure 4B,D). Based on the molecular weight of His-Mms6, its diameter was calculated to be approximately 2.6 nm (the protein was assumed as a spherical protein), implying that a single layer of protein corona is likely to form on the surface of the magnetite nanoparticles.

Based on the surface area of a single SP35 nanoparticle (ca. 3865.5 nm^2^), approximately 729 His-Mms6 molecules can be adsorbed onto one particle as a monolayer. However, the measurements revealed that only 27.7 µg of His-Mms6 were adsorbed by 2.5 mg of SP35, corresponding to approximately 115 molecules per nanoparticle. Based on this calculation, the surface coverage of His-Mms6 on a single magnetite nanoparticle was approximately 15.7%. One potential explanation for this could be that magnetite nanoparticles easily aggregate in solution at pH values close to their pI, resulting in a reduction in the surface area. In this case, the pH used for the protein coating (ca. pH~8) was indeed close to the pI of magnetite (ca. 6.5–7.3), resulting in minimal electrostatic repulsive forces and thus, aggregation. However, it is also possible that the aggregation is induced after protein adsorption and coverage, where the charged surface is neutralized by the binding protein. Low surface coverage in our study might also be because of the aggregation caused by magnetic interactions between nanoparticles. Beyond this, the conformation of the proteins after adsorption may change, thus reducing the number of available contact points on the magnetite surface.

### 2.4. Effect of Crystal Faces on Adsorption of Mms6 on Magnetite Nanoparticle

Mms6 was considered to recognize the (100) facets of the magnetite nanoparticles, resulting in the control of the crystal morphology within the magnetotactic bacterial cells [32]. Therefore, the elucidation of facet specificity is necessary to understand the mechanism of formation of the magnetite nanoparticles in *M. magneticum* AMB-1. To investigate the facet-specificity of His-Mms6, an adsorption assay using magnetite nanoparticles of different sizes and shapes was carried out.

**Figure 5:**
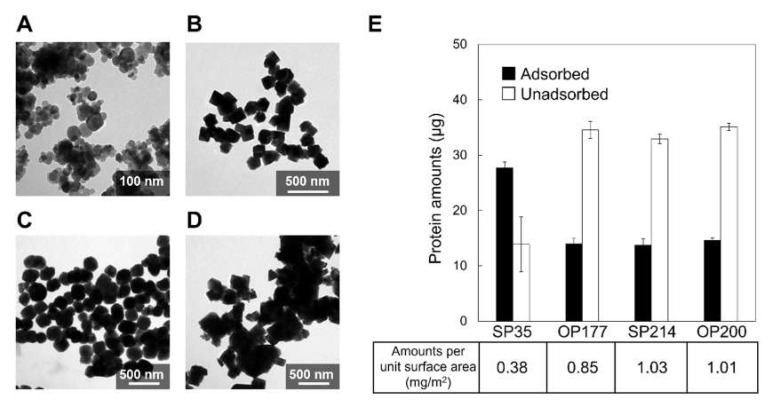
The TEM images of (**A**) SP35, (**B**) OP177, (**C**) SP214, and (**D**) OP200. (**E**) Adsorbed (black bar) or unadsorbed (white bar) protein amounts of His-Mms6 to different magnetite nanoparticles (SP35, OP177, SP214, and OO200). A solution containing 40 µg of protein was added to 2.5 mg of the particles.

For this assay, four types of magnetite nanoparticles were utilized: SP35, spherical nanoparticles with a mean diameter of 35.1 ± 12 nm (Figure 5A); OP177, octahedral nanoparticles with an average diameter of 177.8 ± 36 nm (Figure 5B); SP214, spherical nanoparticles with an average diameter of 214.6 ± 39 nm (Figure 5C); and OP200, octahedral nanoparticles with an average diameter of 200.7 ± 72 nm (Figure 5D). While the amount of His-Mms6 adsorbed on SP35 particles was the greatest at 27.7 µg, the adsorbed amounts of His-Mms6 on the OP177, SP214, and OP200 magnetite nanoparticles were 14.0, 13.7, and 14.6 µg, respectively (Figure 5E). The adsorbed amounts per unit surface area (Figure 5E) were similar between SP214 (1.03 mg/m^2^) and OP200 (1.01 mg/m^2^), which have different morphologies. On the other hand, significantly lower adsorbed amounts of Mms6 per unit surface area occurred on SP35 (ca. 0.38 mg/m^2^). The small sizes of SP35 might account for the low absorption. The TEM images (Figure 5) showed an aggregation of SP35, which lowered the exposed surface area and decreased their adsorption capacity. Furthermore, according to our TEM observations (Figure 5), SP35 was the only nanoparticle in this study that did not have significantly large facets. Thus, the absence of large facets on the nanoparticles may cause a reduction in the adsorption amounts.

### 2.5. Role of Adsorption in the Magnetite Biomineralization by Magnetotactic Bacteria

Magnetotactic bacteria synthesize highly controlled single crystalline magnetite nanoparticles within the internal lipid vesicles. The proteins associated with these vesicles are involved in the crystal formation of magnetite nanoparticles, particularly in the shape and size regulation [32]. Previously, binding studies of Mms6 were conducted using ferrous and ferric ions [28,30,48]. These studies hypothesized that Mms6 functions in the nucleation process of iron oxide crystals. In contrast, Mms6-deficient mutants synthesized smaller magnetite crystals with uncommon crystal faces compared to the wild-type strain [27], suggesting that Mms6 is a key factor in crystal growth rather than nucleation. In addition, Mms6 localizes to the vesicles under magnetite-forming (microaerobic or iron-sufficient) conditions, either before crystal nucleation or during crystal growth [54]; this observation provides a rationale for the function of Mms6 during the crystal growth stage. The direct adsorption of Mms6 onto the magnetite nanoparticles was shown in the present study. According to these observations, Mms6 is adsorbed on magnetite crystals during the growth stage and may invite iron ions needed for its growth on the magnetite crystal surfaces. Our previous report indicated that Mms6 stabilized the (100) facets [32]; however, specific adsorption of Mms6 to the (100) facet of the magnetite nanoparticles was not investigated in this study. Therefore, Mms6 is considered to be involved in both the growth promotion and retardation by ion invitation and facet stabilization through facet-independent adsorption. As previously mentioned, Mms6 changes its conformation from an α-helix to β-sheet in the presence of iron ions (Figure S1). This fact also suggests that Mms6 changes its function such as ion acquisition and adsorption to magnetite via self-assembly, depending on the surrounding environments (i.e., iron ion concentration). However, since there is no experimental evidence supporting the hypothetical molecular function of Mms6, further investigation on the adsorption specificity and functional analysis is necessary.

### 2.6. Adsorption on Inorganic Oxide Particles

**Figure 6:**
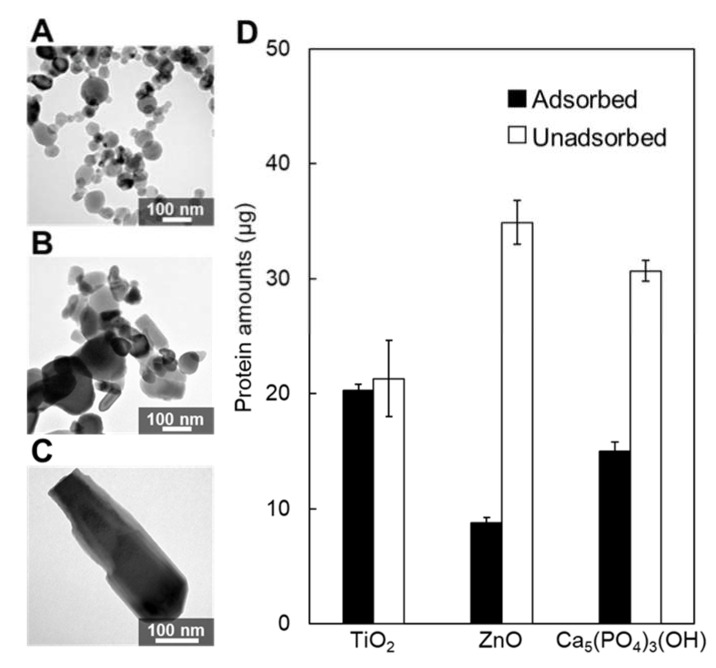
The binding assay of the His-Mms6 protein to nanoparticles. The TEM image of the (**A**) titanium oxide nanoparticles, (**B**) zinc oxide nanoparticles, and (**C**) hydroxyapatite nanoparticles. (**D**) Adsorbed (black bar) or unadsorbed (white bar) protein amounts of His-Mms6 to each nanoparticle. A solution containing 40 µg of protein was added to 2.5 mg of the particles.

In the present study, the adsorption of Mms6 with a high affinity was demonstrated by different assays. To demonstrate the adsorption specificity of Mms6 to magnetite, adsorption studies were carried out on different inorganic oxide particles including titanium oxide (TiO_2_) (Figure 6A), zinc oxide (ZnO) (Figure 6B), and hydroxyapatite [Ca_5_(PO_4_)_3_(OH)] (Figure 6C). These inorganic oxides are prime materials used in the fabrication of biomaterials in medical applications. Using the same assay process with magnetite, the adsorption capacities of Mms6 on titanium oxide, zinc oxide, and hydroxyapatite nanoparticles were 20.1, 8.8, and 15.0 µg, respectively (Figure 6D). The selectivity of Mms for magnetite was not demonstrated in this study; however, Mms6 has demonstrated adsorption on a broad variety of metal oxides.

Combining proteins with nanoparticles yields potentially new functional materials with novel properties for a broad range of applications [55]. In the past few decades, iron magnetic nanoparticles have attracted great attention in the fields of nanomedicine, catalysis, and biomedical applications including drug/gene delivery, nanosensors, and hyperthermia [56]. Mms6, one of the biomineralization proteins reported in this study, has been found to directly adsorb on magnetite nanoparticles and thus can easily be functionalized. Moreover, Mms6 interacted with titanium oxide, zinc oxide, and hydroxyapatite nanoparticles, which highlights the potential of Mms6 in nanoparticle functionalization for nanomedicine and biomedical applications via the direct immobilization of functional proteins on the surfaces of many inorganic nanoparticles.

## 3. Materials and Methods

### 3.1. Materials

For the adsorption assays, we used four inorganic oxide nanoparticles: magnetite (TODA KOGYO CORP, Hiroshima, Japan), titanium oxide (Sigma-Aldrich, St. Louis, MO, USA)_,_ zinc oxide (Sigma-Aldrich, St. Louis, MO, USA), and hydroxyapatite (Sigma-Aldrich, St. Louis, MO, USA). Before each assay, the nanoparticles were washed with phosphate buffer (50 mM KH_2_PO_4_, 300 mM NaCl, 10% glycerol, 0.1% n-dodecyl-β-D-maltoside (DDM), 300 mM imidazole, pH 8.0) and recovered by centrifugation at 10,000× *g* for 10 min. Commercially purchased BSA (Sigma-Aldrich, St. Louis, MO, USA) was dissolved in phosphate buffer at the desired concentrations.

### 3.2. Purification of Recombinant Proteins Expressed in Escherichia coli

Recombinant Mms6, Mms7, and α-synuclein were expressed as a fusion protein with an N-terminal 6x histidine tag in *E. coli* strain BL21 (DE3). The plasmids harboring the *mms6*, *mms7*, and *α-synuclein* genes were constructed by cloning the PCR products into the expression vector pET-15b. Both mms6 and mms7 were amplified from AMB-1 genomic DNA, as described previously [26,32]. The *E. coli* BL21 (DE3) transformants were cultured in LB broth containing 50 µg/mL ampicillin at 37 °C under 1 mM isopropyl-β-D-thiogalactopyranoside induction. The cells were collected by centrifugation and stored at −80 °C until use. The cells were resuspended in lysis buffer (50 mM KH_2_PO_4_, 10% glycerol, and 2% DDM, pH 7.5) with the cOmplete™ protease inhibitor cocktail (Sigma-Aldrich, MO, USA) and homogenized using a French press at 1500 kg/cm^3^ three times. After removing the debris, the supernatant was centrifuged at 100,000× *g* to remove the insoluble proteins. The supernatant was supplemented with 300 mM NaCl and 10 mM imidazole, and incubated with Ni-NTA agarose (QIAGEN, Hilden, Germany) with gentle shaking at 4 °C for 1 h. The resin was pre-equilibrated with 50 mM KH_2_PO_4_ (pH 8.0) containing 10% glycerol, 300 mM NaCl, 10 mM imidazole, and 0.1% DDM. The resin containing the bound protein was then packed into a gravity flow column, and unbound proteins were removed by washing with the buffer used for pre-equilibration. Subsequently, the resin was washed with a buffer containing 30 mM imidazole and His-Mms6, His-Mms7, and His-α-synuclein were eluted with phosphate buffer (50 mM KH_2_PO_4_, 300 mM NaCl, 10% glycerol, 0.1% DDM, and 300 mM imidazole, pH 8.0). Protein concentrations were determined using a BCA Protein Assay Reagent Kit (Thermo Fisher Scientific, Waltham, MA, USA) with bovine serum albumin as the standard. Purified samples were analyzed by SDS-PAGE and the proteins were stained with Bio-Safe Coomassie G-250 (Bio-Rad, Hercules, CA, USA).

### 3.3. In Vitro Protein Adsorption Assay Using Metal Oxide Nanoparticles

#### 3.3.1. Preparation of Protein Solutions

Solutions containing each protein (His-Mms6, His-Mms7, His-α-synuclein, BSA, cytochrome *c*, and lysozyme) were prepared for the in vitro adsorption assays. For the preparation of Mms6, Mms7, and α-synuclein, the recombinant expression system in *E. coli* was used, as described above. Finally, these proteins were eluted from the Ni-NTA agarose resin using phosphate buffer (50 mM KH_2_PO_4_, 300 mM NaCl, 10% glycerol, 0.1% DDM, and 300 mM imidazole, pH 8.0) and stored in the same buffer. The other proteins (BSA, cytochrome *c*, and lysozyme) were commercially purchased as solid powders, and dissolved in phosphate buffer to maintain similar solution conditions.

#### 3.3.2. Protein Adsorption on Metal Oxide Nanoparticles

A solution containing 40 µg of protein was added to 2.5 mg of inorganic oxide nanoparticles in 1.5 mL microtubes, and the suspended mixture was briefly sonicated to disperse inorganic oxide nanoparticles in solution. The mixture was then shaken in a bioshaker (Taitec, Saitama, Japan) overnight at room temperature for protein adsorption on the particles. The supernatants, defined as the unadsorbed fraction, were then separated by centrifugation at 10,000× *g* for 10 min. Finally, the protein contents in the unadsorbed fractions were quantified by the BCA method.

#### 3.3.3. Protein Desorption from Metal Oxide Nanoparticles

A protein desorption experiment was performed to investigate the amount of protein adsorbed on the surfaces of the inorganic oxide nanoparticles. After the removal of the unbound fraction, 100 µL of 1% SDS solution was added to the residual particles and the mixture was boiled at 100 °C for 15 min. The solution was then sonicated in a water bath at room temperature for 15 min. The supernatant solutions, defined as the adsorbed fraction, were separated by centrifugation at 10,000× *g* for 10 min. Finally, the amounts of protein contained in the adsorbed fraction were quantified using the BCA assay.

### 3.4. TEM Analysis

To prepare the samples for TEM, 1 mg of each inorganic oxide nanoparticle was added to 10 mL of ultrapure water and sonicated several times. An aliquot of 5 µL of the solution was deposited, dropwise, onto the carbon-coated TEM copper grids and allowed to dry. The TEM images were collected using a JEOL JEM1200EX (JEOL, Tokyo, Japan) at 120 kV. Another set of grids with iron oxide nanoparticles were stained with 1% uranyl acetate solution for 10 min, followed by rinsing with deionized water three times and drying with filter paper. The samples were further stained with 0.1% lead citrate for 60 s in a CO_2_-free environment by putting NaOH pellets in the staining chamber. The (HR)TEM micrographs of the stained samples were collected with an FEI Titan Themis 300 at 300 kV (Thermo Fisher Scientific, Waltham, MA, USA).

## 4. Conclusions

In summary, the adsorption properties of the magnetite biomineralization proteins (Mms6 and Mms7) on the magnetite nanoparticles were investigated. Consequently, this is the first report that experimentally showed a direct high-affinity (Kd = 9.52 µM) adsorption of Mms6 on the magnetite nanoparticles. Moreover, Mms6 was also adsorbed on the titanium oxide, zinc oxide, and hydroxyapatite nanoparticles. Therefore, Mms6 is able to bind to various solid inorganic materials and have potential in nanoparticle functionalization for nanomedicine and biomedical applications.

## Figures and Tables

**Table 1:** List of the proteins used for the adsorption assay.

Protein	Size (kDa)	pI	Origin
His-Mms6	6.9	6.0	*M. magneticum* AMB-1
His-Mms7	6.5	6.8	*M. magneticum* AMB-1
His-α synuclein	15.5	5.2	Human
BSA	66.3	4.9	Bovine serum

## Supplementary Materials

The following supporting information can be downloaded at: https://www.mdpi.com/article/10.3390/ijms23105554/s1.

## References

[1] Mann S., Hannington J.P., Williams R. (1986). Phospholipid vesicles as a model system for biomineralization. Nature.

[2] Aizenberg J., Black A.J., Whitesides G.M. (1999). Control of crystal nucleation by patterned self-assembled monolayers. Nature.

[3] Weaver J.C., Wang Q., Miserez A., Tantuccio A., Stromberg R., Bozhilov K.N., Maxwell P., Nay R., Heier S.T., DiMasi E. (2010). Analysis of an ultra hard magnetic biomineral in chiton radular teeth. Mater. Today.

[4] Weaver J.C., Milliron G.W., Miserez A., Evans-Lutterodt K., Herrera S., Gallana I., Mershon W.J., Swanson B., Zavattieri P., DiMasi E. (2012). The stomatopod dactyl club: A formidable damage-tolerant biological hammer. Science.

[5] Nemoto M., Wang Q., Li D., Pan S., Matsunaga T., Kisailus D. (2012). Proteomic analysis from the mineralized radular teeth of the giant Pacific chiton, *Cryptochiton stelleri* (Mollusca). Proteomics.

[6] Wang Q., Nemoto M., Li D., Weaver J.C., Weden B., Stegemeier J., Bozhilov K.N., Wood L.R., Milliron G.W., Kim C.S. (2013). Phase transformations and structural developments in the radular teeth of *Cryptochiton stelleri*. Adv. Funct. Mater..

[7] Kisailus D., Nemoto M., Matsunaga T., Tanaka T., Kisailus D. (2018). Structural and proteomic analyses of iron oxide biomineralization in chiton teeth. Biological Magnetic Materials and Applications.

[8] Nemoto M., Ren D., Herrera S., Pan S., Tamura T., Inagaki K., Kisailus D. (2019). Integrated transcriptomic and proteomic analyses of a molecular mechanism of radular teeth biomineralization in *Cryptochiton stelleri*. Sci. Rep..

[9] Huang W., Shishehbor M., Guarín-Zapata N., Kirchhofer N.D., Li J., Cruz L., Wang T., Bhowmick S., Stauffer D., Manimunda P. (2020). A natural impact-resistant bicontinuous composite nanoparticle coating. Nat. Mater..

[10] Wang T., Huang W., Pham C.H., Murata S., Herrera S., Kirchhofer N.D., Arkook B., Stekovic D., Itkis M.E., Goldman N. (2022). Mesocrystalline ordering and phase transformation of iron oxide biominerals in the ultrahard teeth of *Cryptochiton stelleri*. Small Struct..

[11] Hoang Q.Q., Sicheri F., Howard A.J., Yang D.S.C. (2003). Bone recognition mechanism of porcine osteocalcin from crystal structure. Nature.

[12] He G., Dahl T., Veis A., George A. (2003). Nucleation of apatite crystals in vitro by self-assembled dentin matrix protein 1. Nat. Mater..

[13] Lakshminarayanan R., Kini R.M., Valiyaveettil S. (2002). Investigation of the role of ansocalcin in the biomineralization in goose eggshell matrix. Proc. Natl. Acad. Sci. USA.

[14] Brutchey R.L., Yoo E.S., Morse D.E. (2006). Biocatalytic synthesis of a nanostructured and crystalline bimetallic perovskite-like barium oxofluorotitanate at low temperature. J. Am. Chem. Soc..

[15] Sumerel J.L., Yang W., Kisailus D., Weaver J.C., Choi J.H., Morse D.E. (2003). Biocatalytically templated synthesis of titanium dioxide. Chem. Mater..

[16] Kisailus D., Choi J.H., Weaver J.C., Yang W., Morse D.E. (2005). Enzymatic synthesis and nanostructural control of gallium oxide at low temperature. Adv. Mater..

[17] Curnow P., Bessette P.H., Kisailus D., Murr M.M., Daugherty P.S., Morse D.E. (2005). Enzymatic synthesis of layered titanium phosphates at low temperature and neutral pH by cell-surface display of silicatein-α. J. Am. Chem. Soc..

[18] Wang K., Wang X., Li H., Zheng S., Ren Q., Wang Y., Niu Y., Li W., Zhou X., Zhang L. (2018). A statherin-derived peptide promotes hydroxyapatite crystallization and in situ remineralization of artificial enamel caries. RSC Adv..

[19] Blakemore R. (1975). Magnetotactic bacteria. Science.

[20] Sakaguchi T., Burgess J.G., Matsunaga T. (1993). Magnetite formation by a sulphate-reducing bacterium. Nature.

[21] Islam T., Peng C., Ali I. (2018). Morphological and cellular diversity of magnetotactic bacteria: A review. J. Basic Microbiol..

[22] Ozawa E. (2019). Microwave-assisted magnetization reversal in dispersed nanosized barium ferrite particles for high-density magnetic recording tape. IEEE Trans. Magn..

[23] Zhang H.-w., Liu Y., Sun S.-h. (2010). Synthesis and assembly of magnetic nanoparticles for information and energy storage applications. Front. Phys. China.

[24] Matsunaga T., Sakaguchi T., Tadakoro F. (1991). Magnetite formation by a magnetic bacterium capable of growing aerobically. Appl. Microbiol. Biotechnol..

[25] Arakaki A., Yamagishi A., Fukuyo A., Tanaka M., Matsunaga T. (2014). Co-ordinated functions of Mms proteins define the surface structure of cubo-octahedral magnetite crystals in magnetotactic bacteria. Mol. Microbiol..

[26] Arakaki A., Webb J., Matsunaga T. (2003). A novel protein tightly bound to bacterial magnetic particles in *Magnetospirillum magneticum* strain AMB-1. J. Biol. Chem..

[27] Tanaka M., Mazuyama E., Arakaki A., Matsunaga T. (2011). Mms6 protein regulates crystal morphology during nano-sized magnetite biomineralization in vivo. J. Biol. Chem..

[28] Rawlings A.E., Bramble J.P., Hounslow A.M., Williamson M.P., Monnington A.E., Cooke D.J., Staniland S.S. (2016). Ferrous iron binding key to Mms6 magnetite biomineralisation: A mechanistic study to understand magnetite formation using pH titration and NMR spectroscopy. Chem. Eur. J..

[29] Feng S., Wang L., Palo P., Liu X., Mallapragada S.K., Nilsen-Hamilton M. (2013). Integrated self-assembly of the Mms6 magnetosome protein to form an iron-responsive structure. Int. J. Mol. Sci..

[30] Wang L., Prozorov T., Palo P.E., Liu X., Vaknin D., Prozorov R., Mallapragada S., Nilsen-Hamilton M. (2012). Self-assembly and biphasic iron-binding characteristics of Mms6, a bacterial protein that promotes the formation of superparamagnetic magnetite nanoparticles of uniform size and shape. Biomacromolecules.

[31] Prozorov T., Mallapragada S.K., Narasimhan B., Wang L., Palo P., Nilsen-Hamilton M., Williams T.J., Bazylinski D.A., Prozorov R., Canfield P.C. (2007). Protein-mediated synthesis of uniform superparamagnetic magnetite nanocrystals. Adv. Funct. Mater..

[32] Amemiya Y., Arakaki A., Staniland S.S., Tanaka T., Matsunaga T. (2007). Controlled formation of magnetite crystal by partial oxidation of ferrous hydroxide in the presence of recombinant magnetotactic bacterial protein Mms6. Biomater..

[33] Arakaki A., Masuda F., Amemiya Y., Tanaka T., Matsunaga T. (2010). Control of the morphology and size of magnetite particles with peptides mimicking the Mms6 protein from magnetotactic bacteria. J. Colloid Interface Sci..

[34] Bird S.M., Rawlings A.E., Galloway J.M., Staniland S.S. (2016). Using a biomimetic membrane surface experiment to investigate the activity of the magnetite biomineralisation protein Mms6. RSC Adv..

[35] Källberg M., Wang H., Wang S., Peng J., Wang Z., Lu H., Xu J. (2012). Template-based protein structure modeling using the RaptorX web server. Nat. Protoc..

[36] Pyles H., Zhang S., De Yoreo J.J., Baker D. (2019). Controlling protein assembly on inorganic crystals through designed protein interfaces. Nature.

[37] Eguchi Y., Fukumori Y., Taoka A. (2018). Measuring magnetosomal pH of the magnetotactic bacterium *Magnetospirillum magneticum* AMB-1 using pH-sensitive fluorescent proteins. Biosci. Biotechnol. Biochem..

[38] Imamura K., Kawasaki Y., Awadzu T., Sakiyama T., Nakanishi K. (2003). Contribution of acidic amino residues to the adsorption of peptides onto a stainless steel surface. J. Colloid Interface Sci..

[39] Magro M., Cozza G., Molinari S., Venerando A., Baratella D., Miotto G., Zennaro L., Rossetto M., Frömmel J., Kopečná M. (2020). Role of carboxylic group pattern on protein surface in the recognition of iron oxide nanoparticles: A key for protein corona formation. Int. J. Biol. Macromol..

[40] Goobes G., Goobes R., Shaw W.J., Gibson J.M., Long J.R., Raghunathan V., Schueler-Furman O., Popham J.M., Baker D., Campbell C.T. (2007). The structure, dynamics, and energetics of protein adsorption—lessons learned from adsorption of statherin to hydroxyapatite. Magn. Reason. Chem..

[41] Kosmulski M. (2001). Chemical Properties of Material Surfaces.

[42] Peng Z., Hidajat K., Uddin M. (2004). Adsorption of bovine serum albumin on nanosized magnetic particles. J. Colloid Interface Sci..

[43] De Sousa M.E., Fernandez van Raap M.B., Rivas P.C., Mendoza Zélis P., Girardin P., Pasquevich G.A., Alessandrini J.L., Muraca D., Sánchez F.H. (2013). Stability and relaxation mechanisms of citric acid coated magnetite nanoparticles for magnetic hyperthermia. J. Phys. Chem. C.

[44] Dheyab M.A., Aziz A.A., Jameel M.S., Noqta O.A., Khaniabadi P.M., Mehrdel B. (2020). Simple rapid stabilization method through citric acid modification for magnetite nanoparticles. Sci. Rep..

[45] Rehana D., Haleel A.K., Rahiman A.K. (2015). Hydroxy, carboxylic and amino acid functionalized superparamagnetic iron oxide nanoparticles: Synthesis, characterization and in vitro anti-cancer studies. J. Chem. Sci..

[46] Inoue K., Hiradate S., Takagi S. (1993). Interaction of mugineic acid with synthetically produced iron oxides. Soil Sci. Soc. Am. J..

[47] Puddu V., Perry C.C. (2012). Peptide adsorption on silica nanoparticles: Evidence of hydrophobic interactions. ACS Nano.

[48] Zhang H., Liu X., Feng S., Wang W., Schmidt-Rohr K., Akinc M., Nilsen-Hamilton M., Vaknin D., Mallapragada S. (2015). Morphological transformations in the magnetite biomineralizing protein Mms6 in iron solutions: A small-angle X-ray scattering study. Langmuir.

[49] Fincham A., Moradian-Oldak J., Simmer J., Sarte P., Lau E., Diekwisch T., Slavkin H. (1994). Self-assembly of a recombinant amelogenin protein generates supramolecular structures. J. Struct. Biol..

[50] Fincham A., Moradian-Oldak J., Diekwisch T., Lyaruu D., Wright J., Bringas P., Slavkin H. (1995). Evidence for amelogenin" nanospheres" as functional components of secretory-stage enamel matrix. J. Struct. Biol..

[51] Raj P.A., Johnsson M., Levine M.J., Nancollas G.H. (1992). Salivary statherin. Dependence on sequence, charge, hydrogen bonding potency, and helical conformation for adsorption to hydroxyapatite and inhibition of mineralization. J. Biol. Chem..

[52] Bouropoulos N., Moradian–Oldak J. (2003). Analysis of hydroxyapatite surface coverage by amelogenin nanospheres following the Langmuir model for protein adsorption. Calcif. Tissue Int..

[53] Del Pino P., Pelaz B., Zhang Q., Maffre P., Nienhaus G.U., Parak W.J. (2014). Protein corona formation around nanoparticles–from the past to the future. Mater. Horizons.

[54] Arakaki A., Kikuchi D., Tanaka M., Yamagishi A., Yoda T., Matsunaga T. (2016). Comparative subcellular localization analysis of magnetosome proteins reveals a unique localization behavior of Mms6 protein onto magnetite crystals. J. Bacteriol..

[55] Ma W., Saccardo A., Roccatano D., Aboagye-Mensah D., Alkaseem M., Jewkes M., Di Nezza F., Baron M., Soloviev M., Ferrari E. (2018). Modular assembly of proteins on nanoparticles. Nat. Commun..

[56] Liu G., Gao J., Ai H., Chen X. (2013). Applications and potential toxicity of magnetic iron oxide nanoparticles. Small.

