# Supplementary material for: Adsorption of Biomineralization Protein Mms6 on Magnetite (Fe3O4) Nanoparticles
Source: Int J Mol Sci. 2022 May 16;23(10):5554. doi: 10.3390/ijms23105554 (PMC9143127; doi:10.3390/ijms23105554)
Supplement: Supplementary file 1 [file ijms-23-05554-s001.zip › Supplementary Materials.pdf]

Supplementary Materials

Adsorption of biomineralization protein Mms6 on magnetite (Fe_3O_4) nanoparticles

Kosuke Arai[1,†], Satoshi Murata[1,†], Taifeng Wang[2], Wataru Yoshimura[1], Mayumi Oda-Tokuhisa[1], Tadashi Matsunaga[1, 3], David Kisailus[2], Atsushi Arakaki[1, *]

[1]Division of Biotechnology and Life Science, Institute of Engineering, Tokyo University of Agriculture and Technology, 2-24-16 Naka-cho, Koganei, Tokyo 184-8588, Japan; kouok-1030.kej-332@ezweb.ne.jp (K.A.); s-murata@st.go.tuat.ac.jp (S.M.); yosh.wasabi@gmail.com (W.Y.); mayumi.tokuhisa@gmail.com (M.O.-T.); tmatsuna@cc.tuat.ac.jp (T.M.)
[2]Department of Materials Science and Engineering, University of California at Irvine, Irvine, CA 92697, USA; taifengw@uci.edu (T.W.); dkisailu@uci.edu (D.K.)
[3]Japan Agency for Marine-Earth Science and Technology (JAMSTEC), 2-15, Natsushima-cho, Yokosuka, Kanagawa 237-0061, Japan
[*]Correspondence: arakakia@cc.tuat.ac.jp
[†]These authors contributed equally to this work.

Table S1 List of proteins used in this study

Protein	Origin	Size	pI	Amino acid sequence
His-Mms6	*M. magneticum* AMB-1	6.9	6.0	MGHHHHHHGGTIWTGKGLGLGLGLGLGAWGPIILGVVGAGAVYAYMKSRDIESAQSDEEVELRDALA
His-Mms7	*M. magneticum* AMB-1	6.5	6.8	MGHHHHHHAKGLGLGLGLGLGAWGPFLLGAAGLAGAAALYVWARRRHGTPDLSDDALLAAAGEE
His-α synuclein	Human	15.5	5.2	MGHHHHHHMDVFMKGLSKAKEGVVAAAEKTKQGVAEAAGKTKEGVLYVGSKTKEGVVHGVATVAEKTKEQVTNVGGAVVTGVTAVAQKTVEGAGSIAAATGFVKKDQLGKNEEGAPQEGILEDMPVDPDNEAYEMPSEEGYQDYEPEA
BSA	Bovine Serum	66.3	4.9	THKSEIAHRFKDLGEEHFKGLVLIAFSQYLQQCPFDEHVKLVNELTEFAKTCVADESHAGCEKSLHTLFGDELCKVASLRETYGDMADCCEKQEPERNECFLSHKDDSPDLPKLKPDPNTLCDEFKADEKKFWGKYLYEIARRHPYFYAPELLYYANKYNGVFQECCQAEDKGACLLPKIETMREKVLASSARQRLRCASIQKFGERALKAWSVARLSQKFPKAEFVEVTKLVTDLTKVHKECCHGDLLECADDRADLAKYICDNQDTISSKLKECCDKPLLEKSHCIAEVEKDAIPENLPPLTADFAEDKDVCKNYQEAKDAFLGSFLYEYSRRHPEYAVSVLLRLAKEYEATLEECCAKDDPHACYSTVFDKLKHLVDE

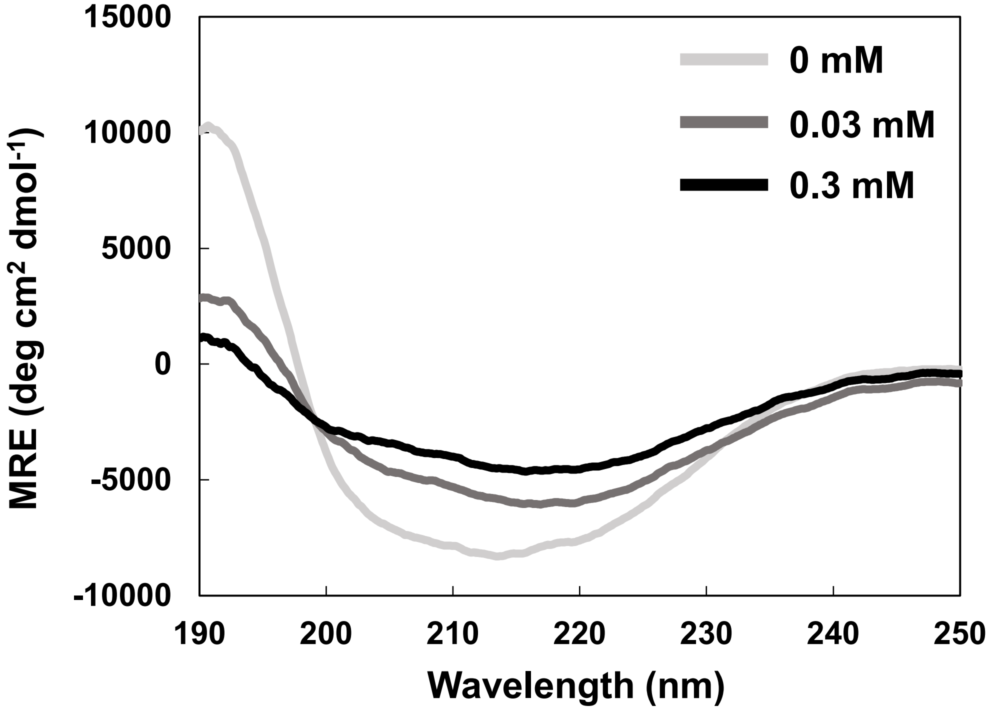

Figure S1 CD spectra of the Mms6 in various Fe^{2+} concentration (0-0.3 mM).
